# *Sox* Gene Family Revealed Genetic Variations in Autotetraploid *Carassius auratus*

**DOI:** 10.3389/fgene.2020.00804

**Published:** 2020-07-28

**Authors:** Xu Huang, Chang Wu, Kaijun Gong, Qian Chen, Qianhong Gu, Huan Qin, Chun Zhao, Tingting Yu, Li Yang, Wen Fu, Yude Wang, Qinbo Qin, Shaojun Liu

**Affiliations:** State Key Laboratory of Developmental Biology of Freshwater Fish, Engineering Research Center of Polyploid Fish Reproduction and Breeding of the State Education Ministry, College of Life Sciences, Hunan Normal University, Changsha, China

**Keywords:** *Sox* gene family, whole genome duplication, autotetraploid, polyploidization, hybridization

## Abstract

The *Sox* gene family encoded transcription factors that played key roles in developmental processes in vertebrates. To further understand the evolutionary fate of the *Sox* gene family in teleosts, the *Sox* genes were comprehensively characterized in fish of different ploidy levels, including blunt snout bream (2n = 48, *Megalobrama amblycephala*, BSB), goldfish (2n = 100, *Carassius auratus* red var., 2nRCC), and autotetraploid *C. auratus* (4n = 200, 4nRCC). The 4nRCC, which derived from the whole genome duplication (WGD) of 2nRCC, were obtained through the distant hybridization of 2nRCC (♀) × BSB (♂). Compared with the 26 *Sox* genes in zebrafish (2n = 50, *Danio rerio*), 26, 47, and 92 putative *Sox* genes were identified in the BSB, 2nRCC, and 4nRCC genomes, respectively, and classified into seven subfamilies (B1, B2, C, D, E, F, and K). Comparative analyses showed that 89.36% (42/47) of *Sox* genes were duplicated in 2nRCC compared with those in BSB, while 97.83% (90/92) of *Sox* genes were duplicated in 4nRCC compared with those in 2nRCC, meaning the *Sox* gene family had undergone an expansion in BSB, 2nRCC, and 4nRCC, respectively, following polyploidization events. In addition, potential gene loss, genetic variations, and paternal parent SNP locus insertion occurred during the polyploidization events. Our data provided new insights into the evolution of the *Sox* gene family in polyploid vertebrates after several rounds of WGD events.

## Introduction

Several rounds of whole genome duplication (WGD) events were known to have taken place during the evolution of vertebrates (Voldoire et al., 2017). The two rounds (2R) of WGD events in vertebrates might be traced back to 500–600 million years ago (Mya), and teleosts were widely believed to have undergone teleost-specific WGD (TSGD or third round, 3R) events approximately 320–350 Mya (Venkatesh, 2003; Guo et al., 2009; Van de Peer et al., 2009; Li et al., 2014; Zhang et al., 2018). The TSGD might have resulted in the wide variety of the teleosts. More than 32,500 species of fish existed in nature, making them the most diverse group of vertebrates (Cossins and Crawford, 2005; Liu, 2014; Wang et al., 2018). Moreover, the common carp (*Cyprinus carpio*) and the goldfish (*Carassius auratus*), which represented the “tetraploid state” in relation to other members of the Cyprinidae family (*Danio rerio*), were thought to have undergone an additional round (fourth round, 4R) of genome duplication (Ohno et al., 1967; Larhammar Dan, 1994; David et al., 2003; Wang et al., 2012; Chen et al., 2019; Xu et al., 2019). Hence, the natural polyploids formed by genome duplication would have subsequently become new diploids or paleopolyploids through diploidization and differentiation (Jenczewski and Alix, 2004; Parisod et al., 2010; Song et al., 2012; Qin et al., 2016; Zhou and Gui, 2017). Genome duplication could thus be considered an important process in species origination and evolution.

The goldfish (2nRCC, 2n = 100) is raised over all Asia for food and as an ornamental pet (Liu et al., 2016; Chen et al., 2019), while *Megalobrama amblycephala* (blunt snout bream, 2n = 48, BSB) is an economically important freshwater fish (Ren et al., 2019). In previous studies, we artificially obtained fertile allotetraploids (4nRB, 4n = 148, F_1_) from the distant hybridization of 2nRCC (♀) × BSB (♂) (Liu et al., 2007). The fertile 4nRCC (4n = 200) was obtained through the fertilization of diploid sperm and eggs caused by the abnormal chromosome behavior during meiosis of 4nRB, and the 4nRCC lineage (F_2_–F_14_) was established through continuous self-crossing (Qin et al., 2014; Hu et al., 2019). The newly synthesized 4nRCC possessed four sets of chromosomes from 2nRCC and produced diploid gametes, and there were obvious phenotypic differences between 2nRCC and 4nRCC (Qin et al., 2014; Qin Q. et al., 2018; Qin Q. B. et al., 2018). It has been stated that studies of recently polyploid fishes and their closely related species might be essential to investigate the evolutionary processes occurring in polyploid genomes (gene families) after polyploidization (Moghadam et al., 2005; Guo et al., 2009). Thus, the newly synthesized 4nRCC might be one of the best model species to study polyploidization and genome duplication.

The *Sox* gene family shared a high mobility group (HMG) box, which contained a DNA-binding domain of 79 amino acids (aa) (Chiang et al., 2001; Yokoi et al., 2002; Hett and Ludwig, 2005). Based on conserved protein domains and nucleic acid sequences, the *Sox* gene family was divided into 11 groups from SoxA to SoxK (Yu et al., 2018). The *Sox* genes were involved in the regulation of diverse growth-related and developmental processes, such as chondrogenesis, neurogenesis, and sex determination and differentiation (Jiang et al., 2013). For example, the *Sox9a* gene presented significant sex correlations in both 4nRCC and 2nRCC (Huang et al., 2020). Currently, extensive studies had identified the *Sox* gene family in various species, and the number of *Sox* genes had been found to vary greatly (Wei et al., 2016; Yu et al., 2017). In teleosts, 19 *Sox* genes were found in Japanese medaka (*Oryzias latipes*, 3R), 24 in pufferfish (*Fugu rubripes*, 3R), 25 in catfish (*Ictalurus punctatus*, 3R) and Japanese flounder (*Paralichthys olivaceus*, 3R), 26 in zebrafish (*D. rerio*, 3R), and 27 in tilapia (*Oreochromis niloticus*, 3R), while 49 putative *Sox* genes were present in common carp (*C. carpio*, 4R) (Cui et al., 2011; Wei et al., 2016; Yu et al., 2018; Zhang et al., 2018). Obviously, from 3R to 4R, more *Sox* genes existed in common carp than in other species, suggesting that most *Sox* genes also underwent expansion. Natural and synthetic polyploid fish might provide ideal models for examining the evolutionary behavior of *Sox* genes in duplicated genomes after polyploidization events (Guo et al., 2009). For the important role of *Sox* gene family in developmental processes in Cyprinidae fishes with different ploidy levels, studies of this family may provide a better understanding the effect of genome duplication.

In this study, we analyzed the *Sox* gene sequences of BSB (2n = 48), 2nRCC (2n = 100), and 4nRCC (4n = 200) compared with those of zebrafish (2n = 50). Based on all the available *Sox* genes of zebrafish, the putative *Sox* gene sequences of BSB and 2nRCC were obtained using the available genome databases of BSB and goldfish, respectively. Subsequently, PCR surveys were performed for *Sox* gene sequences in 4nRCC. This study aimed to perform a comprehensive investigation of the diversity and phylogenetic relationships of the *Sox* gene family in BSB, 2nRCC, and 4nRCC, thus elucidating the genomic effects of polyploidization events.

## Materials and Methods

### Materials

All the individuals of BSB, 2nRCC, and 4nRCC in this paper were cultured in similar pond with area of 666.7 m^2^ in the State Key Laboratory of Developmental Biology of Freshwater Fish, Hunan Normal University, Changsha, Hunan, China. The 4nRCC was produced from the same fish lineage of BSB in this study. The individuals used as samples in this study weighed about 100 g and were anesthetized with 100 mg/L MS-222 (Sigma–Aldrich, St. Louis, MO, United States) prior to dissection.

### Preparation of Chromosome Spreads

In this study, to better understand the changes in chromosome number doubling after genome replication in BSB, 2nRCC, and 4nRCC, chromosome counts were performed using kidney tissue from 10 individuals each of BSB, 2nRCC, and 4nRCC. A detailed method can be found in Liu et al. (2007). Twenty metaphase spreads of chromosomes for each individual were photographed under a microscope.

### Identification of the *Sox* Genes in the BSB and 2nRCC Genomes

All available *Sox* genes were identified in the zebrafish genome^1^. These *Sox* genes were used to search for their respective BSB and 2nRCC counterparts in the available BSB and 2nRCC genomic resources (project ID: PRJNA481500) using Blast (V2.5.0) software (Chen et al., 2019; Ren et al., 2019). The CDs lengths and aa numbers of the putative *Sox* genes were identified from the BSB and 2nRCC genomes.

### RNA Isolation and RT-PCR

According to the spatial expression profiles of the tilapia and Japanese flounder *Sox* genes, most *Sox* genes show high expression in five adult tissues, including brains, testes, ovaries, hearts, and kidneys (Wei et al., 2016; Yu et al., 2018). Hence, total RNA was isolated from the brains, testes, ovaries, hearts, and kidneys tissues of 10 4nRCC individuals using RNAiso reagent (TaKaRa, Japan) following the manufacturer’s protocol. Total RNA integrity was detected by 1.5% agarose gel electrophoresis and a Synergy 2 Multi-Mode Microplate Reader (BioTek^2^), and the samples were stored at −80°C. The first-strand cDNA was synthesized using the Maxima H Minus First Strand cDNA Synthesis Kit with dsDNase (Thermo Scientific, United States) in a 20 μL reaction volume.

### PCR Amplification, Cloning, and Sequencing of 4nRCC *Sox* Genes

In the genetic composition, the 4nRCC were derived from the WGD of 2nRCC (Qin et al., 2014). Based on the CDs of the 2nRCC putative *Sox* genes, primers (Supplementary Table S1) were designed by Primer 5.0 software to amplify 4nRCC *Sox* genes with cDNA as templates. The primers were synthesized by Tsingke (Beijing, China). PCR was performed in a final volume of 50 μL using LA Taq (TaKaRa). The amplification conditions were as follows: 5 min at 94°C; 35 cycles of 30 s at 94°C, 30 s at 50–63°C, and 2 min at 72°C; and 5 min at 72°C for final extension (Wang et al., 2017). All PCR products were cloned into the pMD18-T vector (TaKaRa) and transferred into *Escherichia coli* DH5α (Sangon, China). To avoid PCR errors, at least 60 positive clones for each gene were obtained after screening by PCR amplification and sequenced by Tsingke. All sequences were analyzed using Blast^3^.

### Phylogenetic Analysis, Gene Nomenclature, and Identification of Conserved Motifs

Multiple sequence alignments of the CDs and derived aa sequences of the *Sox* genes in zebrafish, BSB, 2nRCC, and 4nRCC were performed using BioEdit software. Phylogenetic analysis was used to better understand the evolution of *Sox* genes in these species using MrBayes version 3.1.2. The mixed model was chosen for the *Sox* genes as no substitution model could be decided according the aa sequence. MrBayes was run for 10 million generations with two runs and four chains in parallel and a burn-in of 25%, and the analysis was terminated after the average standard deviation of the split frequencies fell under 0.01 (Luo et al., 2019). The final trees were visualized in FIGTREE (V1.4.4) software. Phylogenetic analysis is considered to be one of the most important pieces of evidence for the gene annotation and nomenclature of non-model species (Jiang et al., 2016). All the *Sox* genes from BSB, 2nRCC, and 4nRCC were named after their closely related zebrafish *Sox* genes. When more than one *Sox* gene from 2nRCC or 4nRCC was clustered with certain zebrafish *Sox* genes, Roman-letter suffixes were added to each *Sox* gene (for instance, 2nRCC_*Sox1a*-1, 2nRCC_*Sox1a*-2, 4nRCC_*Sox1a*-1, 4nRCC_*Sox1a*-2, 4nRCC_*Sox1a*-3, 4nRCC_*Sox1a*-4). Generally, Sequence motif is defined as a sequence pattern of DNA, RNA, or aa sequence. Conserved motifs were checked using the online Multiple Expectation Maximization for Motif Elicitation (MEME^4^) program (Zhao et al., 2018).

### Statistical Analysis

Analyses of variance and pairwise comparisons of the data were performed with SPSS 17.0 software.

## Results

### Examination of Chromosome Number

**FIGURE 1:**
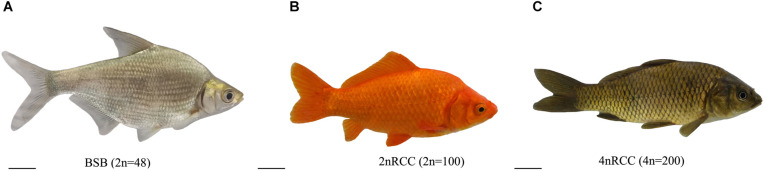
The morphological appearances of BSB **(A)**, 2nRCC **(B)**, and 4nRCC **(C)**. Bar = 1 cm.

**FIGURE 2:**
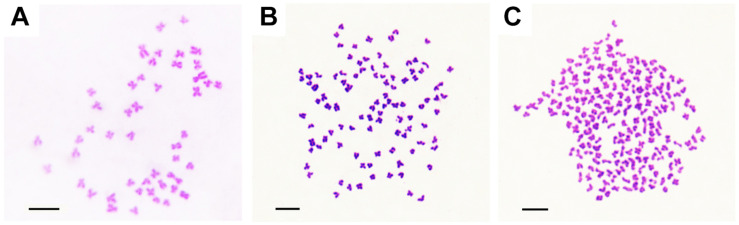
The chromosomes at metaphase in BSB, 2nRCC, and 4nRCC. **(A)** The 48 chromosomes of BSB. **(B)** The 100 chromosomes of 2nRCC. **(C)** The 200 chromosomes of 4nRCC. Bar = 20 μm.

The appearances of BSB, 2nRCC, and 4nRCC were presented in Figure 1. The chromosome number distributions in BSB, 2nRCC, and 4nRCC are shown in Table 1 and Figure 2. In the BSB samples (Figure 1A), 92.5% of the chromosomal metaphases possessed 48 chromosomes (Table 1 and Figure 2A). Among the 2nRCC samples (Figure 1B), 90.5% of the chromosomal metaphases possessed 100 chromosomes (Table 1 and Figure 2B). Among the 4nRCC samples (Figure 1C), 85% of the chromosomal metaphases had 200 chromosomes (Table 1 and Figure 2C), indicating that distant hybridization could result in polyploidization events accompanied by chromosome number doubling.

**TABLE 1:** Examination of the chromosome numbers of BSB, 2nRCC, and 4nRCC.

**Fish type**	**No. in metaphase**	**Distribution of chromosome number**					
		**<48**	**48**	**<100**	**100**	**<200**	**200**
BSB	200	15	185				
2nRCC	200			19	181		
4nRCC	200					30	170

### Identification of *Sox* Genes in BSB, 2nRCC, and 4nRCC

A total of 26 *Sox* genes were identified in the zebrafish genome^1^ (Yu et al., 2018). By searching for these sequences in the BSB and 2nRCC genomes, we identified 26 and 47 putative *Sox* genes in BSB and 2nRCC, respectively (Supplementary Tables S2, S3). In addition, a total of 92 *Sox* genes were identified in 4nRCC by PCR survey (Supplementary Table S4). All the coding sequences (CDs) from 4nRCC were confirmed to be *Sox* gene sequences via the NCBI website database^5^. Through a comprehensive comparison, we noticed that the number of *Sox* genes between BSB and zebrafish were the same, indicating that BSB had also undergone a TSGD event; we also noticed an important expansion of the *Sox* gene family following genome duplication (Table 2): compared with BSB, 89.36% (42/47) of *Sox* genes were duplicated in 2nRCC due to the 4R WGD event; from 2nRCC to 4nRCC, 97.83% (90/92) of *Sox* genes were duplicated due to a specific WGD event. These results indicated that the number of most *Sox* genes in each species were generally associated with ploidy level.

**TABLE 2:** Gene numbers in zebrafish, BSB, 2nRCC, and 4nRCC.

**Gene name**	**Zebrafish**	**BSB**	**2nRCC**	**4nRCC**
*Sox1*	2	2	4	8
*Sox2*	1	1	2	4
*Sox3*	1	1	2	4
*Sox19*	2	2	4	8
*Sox14*	1	1	2	4
*Sox21*	2	2	4	8
*Sox4*	2	2	4	8
*Sox11*	2	2	2	4
*Sox12*	1	1	2	4
*Sox5*	1	1	2	4
*Sox6*	1	1	1	2
*Sox13*	1	1	2	4
*Sox8*	2	2	4	8
*Sox9*	2	2	4	8
*Sox10*	1	1	2	4
*Sox7*	1	1	2	4
*Sox17*	1	1	1	1
*Sox18*	1	1	2	4
*Sox32*	1	1	1	1
Total	26	26	47	92

### SNP Loci From the Paternal Parent in 4nRCC and Sequence Analyses

**FIGURE 3:**
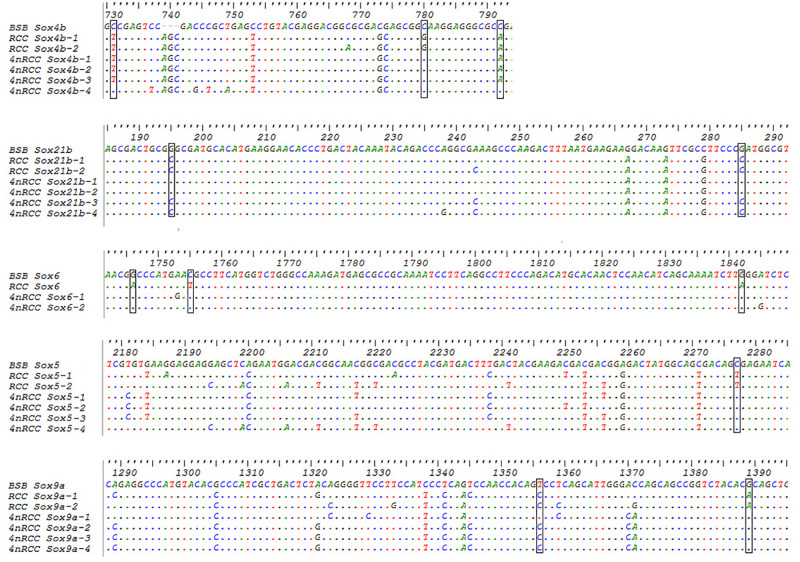
The SNP loci from BSB found in the 4nRCC sequencing results. The dots represented sequence identity, and the hyphens represented insertions/deletions. The black boxes showed the SNP loci.

**FIGURE 4:**
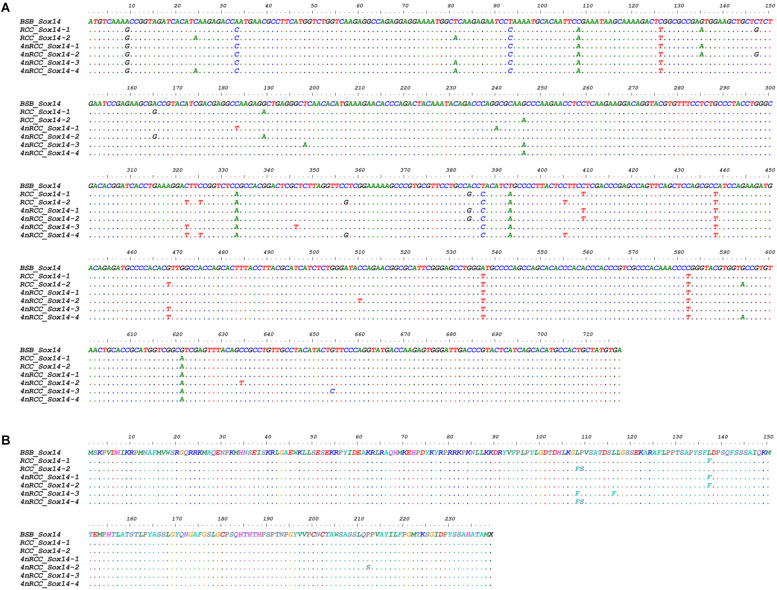
Alignment of the *Sox14* CDs **(A)** and derived aa sequences **(B)** of BSB, 2nRCC, and 4nRCC. The dots represented sequence identity.

**FIGURE 5:**
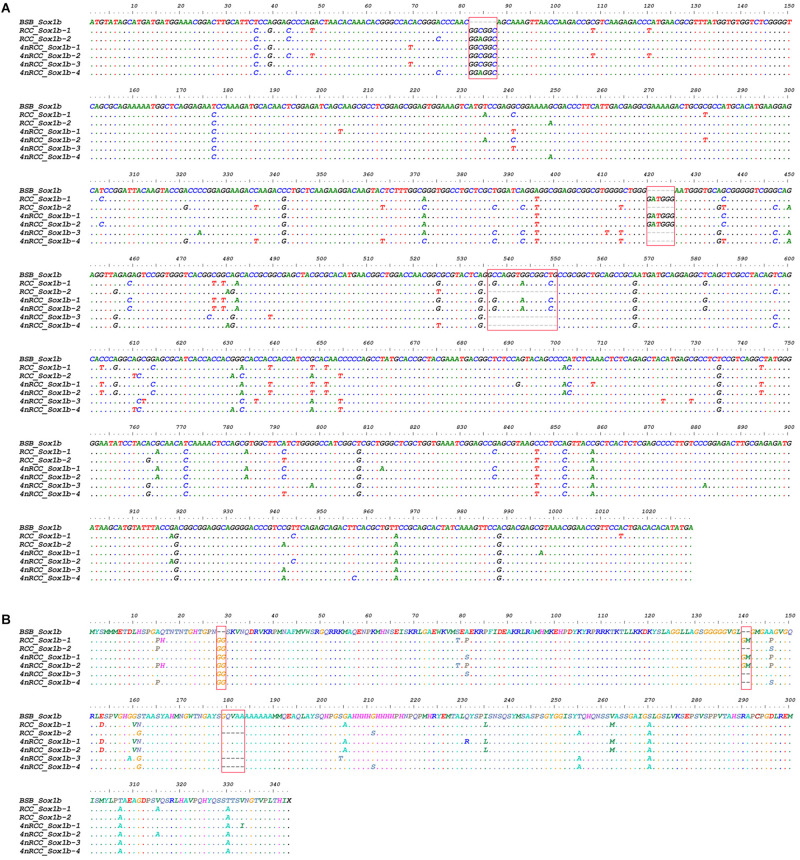
Alignment of the *Sox1b* CDs **(A)** and derived aa sequences **(B)** of BSB, 2nRCC, and 4nRCC. The dots represented sequence identity, and the hyphens represented insertions/deletions. Base insertions/deletions and aa insertions/deletions were framed by red boxes.

We examined the sequence divergence of all *Sox* genes in BSB, 2nRCC, and 4nRCC. The identity for most *Sox* genes between 4nRCC and BSB was lower than that between 4nRCC and 2nRCC. However, as shown in Figure 3, SNP loci were found in the 4nRCC sequencing results that were identical to the BSB sequence but different from that of 2nRCC. According to the multiple alignment, synonymous mutations and several base insertions/deletions were observed in the coding regions of most *Sox* genes among BSB, 2nRCC, and 4nRCC. For instance, some SNP mutations were observed in all *Sox14* genes (Figure 4A), and all *Sox14* genes had higher identities in the aa sequence than in the CDs (Figure 4B), suggesting that most mutations were synonymous. As shown in Figure 5, two six-base insertions/deletions and a 15-base insertion/deletion were found in *Sox1b*; two two-amino-acid insertions/deletions and a five-amino-acid insertion/deletion were found in the corresponding putative aa sequence.

### Phylogenetic Analysis

**FIGURE 6:**
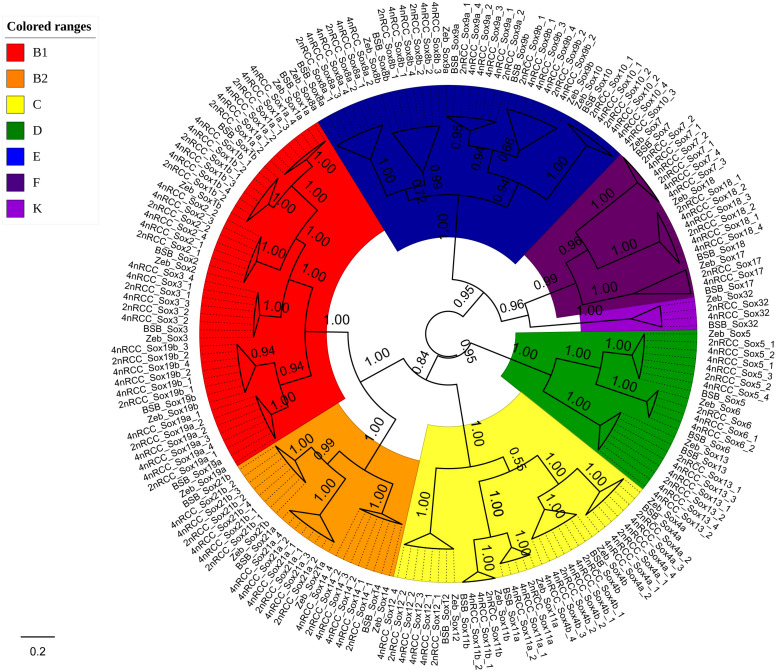
Phylogenetic analyses of the amino acid sequences of all the *Sox* genes in zebrafish (Zeb), BSB, 2nRCC, and 4nRCC. The phylogenetic tree constructed using MrBayes with mixed model; MCMC = 10 million generations. Different branch colors denoted different *S*ox subfamilies.

We further used the putative aa sequences of the *Sox* genes to build phylogenetic trees for zebrafish, BSB, 2nRCC, and 4nRCC (Figure 6). The phylogenetic analysis showed that the *Sox* genes of BSB, 2nRCC, and 4nRCC were clustered with their respective counterparts from zebrafish, providing strong evidence for correctly naming and grouping the *Sox* genes of BSB, 2nRCC, and 4nRCC and indicating that all the genes in the *Sox* gene family were highly conserved. All the putative *Sox* genes could be divided into seven subfamilies. In BSB, six putative genes were clustered in subfamily B1, three in subfamily B2, five in subfamily C, three in subfamily D, five in subfamily E, three in subfamily F, and one in subfamily K. In 2nRCC, 12 putative genes were clustered in subfamily B1, six in subfamily B2, eight in subfamily C, five in subfamily D, 10 in subfamily E, five in subfamily F, and one in subfamily K. In 4nRCC, the PCR survey showed 24 putative genes in subfamily B1, 12 in subfamily B2, 16 in subfamily C, 10 in subfamily D, 20 in subfamily E, nine in subfamily F, and one in subfamily K. The names and numbers of as for each *Sox* gene in BSB, 2nRCC, and 4nRCC are listed in Supplementary Tables S1–S3, respectively. However, some *Sox* genes, such as *Sox17* and *Sox32*, retained only one copy in these four fishes. Some other *Sox* genes, including *Sox6* and *Sox11*, retained their original number and did not undergo duplication (Table 2) after the 4R WGD event.

### Identification of Conserved Motifs

**FIGURE 7:**
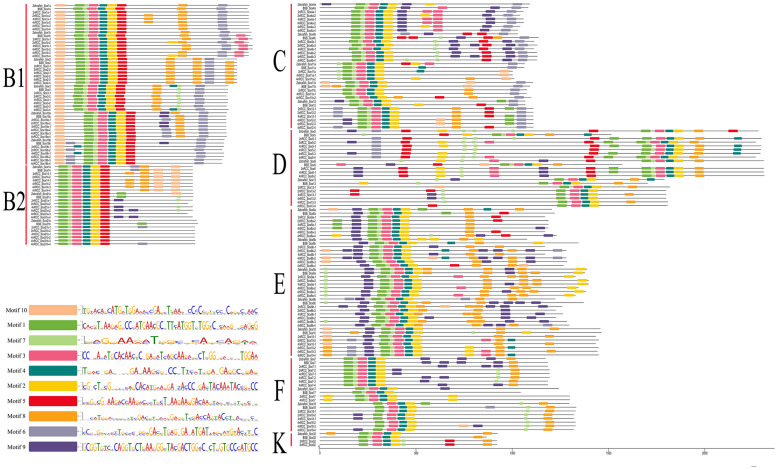
Conserved motifs of seven subfamilies (B1, B2, C, D, E, F, and K) of all *Sox* genes in zebrafish, BSB, 2nRCC, and 4nRCC. Conserved motifs were checked using the online Multiple Expectation Maximization for Motif Elicitation (MEME, http://alternate.meme-suite.org/) program. Different colors denoted different motifs in *Sox* genes, and the sequence of motif 1–10 in *Sox* genes was presented.

A total of 10 conserved motifs (motifs 1–10) are identified in Figure 7. In general, subfamily B1, subfamily B2, subfamily C, subfamily D, and subfamily E had ten motifs (1, 2, 3, 4, 5, 6, 7, 8, 9, 10); subfamily F had nine motifs (1, 2, 3, 4, 6, 7, 8, 9, 10); and subfamily K had seven motifs (1, 2, 3, 4, 5, 7, 8). Comparing these motifs with the conserved HMG box sequence of vertebrate *Sox* proteins (DHVKRPMNAFMVWSRGERRKIAQQNPDMHNSEISKRLG KRWKLLSESEKRPFIEEAERLRAQHMKDYPDYKYRPRRKKK) (Yu et al., 2018), we found that complete motifs 1, 2, 3, 4, and 7 and partial motif 5 constituted the conserved HMG domain. *Sox* genes in the same subfamily shared similar conserved motifs that were clearly distinguishable from those in other subfamilies.

## Discussion

Autopolyploids were traditionally considered to arise by the WGD of a species, whereas allopolyploids were produced by hybridization and the merging of genomes from different species (Julca et al., 2018). Evidence has accumulated that an additional round (4R) of genome duplication in some Cyprinidae species occurred approximately 8–12 Mya, suggesting that those Cyprinidae species, which include goldfish and common carp, are natural allotetraploids (Ma et al., 2014; Wei et al., 2016; Chen et al., 2019; Omori and Kon, 2019; Xu et al., 2019). In previous study, we successfully obtained the synthetic line 4nRCC, which was derived from a specific WGD event of 2nRCC induced by distant hybridization (Qin et al., 2014). In this study, we noticed an important expansion of the *Sox* gene family in 4nRCC (4n = 200): 97.83% (90/92) of *Sox* genes were duplicated compared with 2nRCC (2n = 100). Similarly, the duplication rate of *Sox* genes in 2nRCC reached 89.36% (42/47) compared with those in BSB (3R, 2n = 48), while the number of chromosomes in 2nRCC was approximately twice that in BSB. Hence, our findings also provided evidence that 2nRCC was a tetraploid and had undergone 4R WGD.

Lineage-specific gene duplication and gene loss events were frequently observed in fish genomes during evolution; these events represented one of the primary driving forces for genome evolution and provided useful information for gene evolution studies (Fortna et al., 2004; Jiang et al., 2016; Zhang et al., 2018; Han et al., 2019). Gene duplication was the key mechanism for generating new genes and biological processes, which would be conducive to the evolution of species (Fortna et al., 2004). Moreover, a “1-2-4 (-8 in teleosts)” rule had been proposed for the evolution of vertebrate gene and genome duplications (Axel Meyer, 1999; Meyer and Van de Peer, 2005). In this study, all duplicated genes, except for *Sox6*, *Sox11*, *Sox17*, and *Sox32*, were consistent with this proposal, creating new homologous *Sox* genes following the genome duplication. For instance, there were four *Sox19a* genes in 4nRCC (artificial tetraploid, 4n = 200), two *Sox19a* genes in 2nRCC (diploidized tetraploid, 2n = 100), and only one in BSB (diploid, 2n = 48). In addition, phylogenetic analyses and identification of conserved motifs demonstrated that all *Sox* genes were conserved. Following the emergence of new genes, the regulatory networks during the development of vertebrates might become more complicated to cope with the complex and ever-changing natural environment (Kondrashov, 2012). The specific functions of *Sox* genes in 4nRCC and 2nRCC remain unclear, which need to be further investigated. Thus, our findings also served to support the “1-2-4 (-8 in teleost fishes)” rule in natural and synthetic polyploid fishes. According to this rule and the numbers of most of the duplicated *Sox* genes, we speculated that the newly synthesized 4nRCC might in fact be octoploid. Of course, further experiments are required to verify this hypothesis.

Similar duplication events were also observed in other gene families. For example, there were at least 16 FGFs in common carp (4R) with duplicated copies comparable to the eight FGFs in zebrafish (3R) (Jiang et al., 2016). A special result was also found in sturgeons that seven *Hox* clusters including 68 *Hox* genes were identified (Cheng et al., 2019). Although the sturgeons did not undergo the teleost-specific genome duplication (TSGD) event, they have experienced their own lineage-specific polyploidizations with one or more rounds of genome duplication (Jaillon et al., 2004; Crow et al., 2012). We speculated that the similar duplication events might also occur in other gene families in 4nRCC.

Some *Sox* genes (*Sox6*, *Sox11*, *Sox17*, and *Sox32*) retained their original number and did not undergo duplication in the 4R WGD or specific WGD. Potential gene loss were also observed in other gene families, such as the Fibroblast growth factors (FGFs) in common carp (4R) and the ATP-binding cassette (ABC) transporter gene superfamily in catfish (3R) (Liu et al., 2013; Jiang et al., 2016). Although a genome duplication should be accompanied by the doubling of all genes, gene losses might occur in the state of natural or synthetic polyploidy following WGD events for reasons of critical selective pressure; organisms may need to overcome genomic incompatibility through genomic changes. It is worth noting that imperfect genome assembly and annotation may also be one of the reasons for “gene loss,” especially on polyploids that have recently experienced WGD events (Jiang et al., 2016).

Interestingly, deletions and mutations were also observed in the coding regions of most *Sox* genes, indicating that WGD events could lead to genomic sequence diversity and that genomic alterations were part of adapting to polyploidization. Although 4nRCC was derived from a specific WGD event of 2nRCC induced by distant hybridization, SNP loci derived from the paternal parent were also detected in 4nRCC. In previous studies, chimeric SNP loci (or chimeric genes) were also detected in other hybrid lineage, which might produce structural changes by affecting normal transcriptional processing (Liu et al., 2016; Wu et al., 2020). Hence, we speculated that the expansion of diversity in the *Sox* gene family further revealed that distant hybridization as a catalyst accelerated the process of polyploidization and speciation, and these processes in polyploid formation provided more insight into the origins of natural and synthetic polyploid vertebrates.

## Conclusion

This study revealed an important expansion of the *Sox* gene family in Cyprinidae fishes with different ploidy levels and represented a comparative analysis of natural and synthetic polyploids. In this study, a total of 26, 47, and 92 putative *Sox* genes were identified in the BSB, 2nRCC, and 4nRCC genomes, respectively. Comparative analyses revealed that the *Sox* gene family had undergone duplication in Cyprinidae fishes following WGD events. Multiple alignment, phylogenetic, and conserved motif analyses revealed that most of the *Sox* genes were well conserved during evolution. Our data provided valuable information about genome duplication and new insights into the expansion and evolution of *Sox* genes in polyploid vertebrates after several rounds of WGD events; these findings also suggested that distant hybridization was an effective method to accelerate rates of speciation or evolution.

## Data Availability Statement

The datasets presented in this study can be found in online repositories. The names of the repository/repositories and accession number(s) can be found in the article/Supplementary Material.

## Ethics Statement

Fish treatments were carried out according to the regulations for protected wildlife and the Administration of Affairs Concerning Animal Experimentation and approved by the Science and Technology Bureau of China. Approval from the Department of Wildlife Administration was not required for the experiments conducted in this manuscript.

## Author Contributions

SL, QQ, and XH designed the study. XH provided the preliminary data that supported this study and wrote the manuscript. CW and KG performed the bioinformatics analysis. XH, CW, and KG participated in phylogenetic analysis and discussions. QC, HQ, CZ, and TY participated in the sequence alignment. LY, WF, and YW prepared the chromosome spreads. SL, QQ, and CW provided expert comments. QG also participated in phylogenetic analysis and reconstructed the phylogenetic tree. All authors read and approved the final manuscript.

## Conflict of Interest

The authors declare that the research was conducted in the absence of any commercial or financial relationships that could be construed as a potential conflict of interest.

## Footnotes

1 http://asia.ensembl.org/index.html

2 www.biotek.com

3 https://blast.ncbi.nlm.nih.gov/Blast.cgi

4 http://alternate.meme-suite.org/

5 http://www.ncbi.nlm.nih.gov

## Supplementary Material

TABLE S1 PCR primers designed based on the putative CDs of the goldfish *Sox* genes.

TABLE S2 Inventory of the *Sox* genes in BSB.

TABLE S3 Inventory of the *Sox* genes in 2nRCC.

TABLE S4 Inventory of the *Sox* genes in 4nRCC.

## Abbreviations

2nRCC: *Carassius auratus* red var.
4nRCC: autotetraploid *Carassius auratus*
BSB: *Megalobrama amblycephala*
CDs: coding sequences
SNP: single nucleotide polymorphism.
