# Supplementary material for: Sox Gene Family Revealed Genetic Variations in Autotetraploid Carassius auratus
Source: Front Genet. 2020 Jul 28;11:804. doi: 10.3389/fgene.2020.00804 (PMC7399338; doi:10.3389/fgene.2020.00804)
Supplement: TABLE S1 — PCR primers designed based on the putative CDs of the goldfish Sox genes. [file Data_Sheet_1.DOCX]

**Table S1.** PCR primers designed based on the putative CDs of the goldfish *Sox* genes.

| **Gene** | **Forward primer** | **Reverse primer** |
| --- | --- | --- |
| *Sox1a-1* | ATGTATAGCATGATGATGGAAACG | TCAAATATGCGTCAATGGGAC |
| *Sox1a-2* |  | TCAAATATGCGTCAGTGGGAC |
| *Sox1b* | ATGTATAGCATGATGATGGAAACG | TCATATGTGTGTCAGTGGAACGG |
| *Sox2-1* | ATGTATAACATGATGGAAACCGAGC | TTACATGTGTGATAAGGGGATGG |
| *Sox2-2* | ATGTATAACATGATGGAGACCGAGC | TTACATGTGTGATAAGGGCATGG |
| *Sox3-1* | ATGTATAGCATGATGGAAACCGAG | TCAAATGTGGGTTAGGGGTAGC |
| *Sox3-2* | ATGTATAACATGATGGAAACCGAG | TCAGATGTGGGTCAGGGGTAGC |
| *Sox19a-1* | ATGTACAGCATGCTGGAACACG | TCAGATGTGAGTGAGAGGTGCTG |
| *Sox19a-2* |  | TCAGATGTGGGTGAGAGGTGCTG |
| *Sox19b* | ATGTACAGCATGATGGAG | TCAGATATGAGTGAGGGGAACAG |
| *Sox14* | ATGTCAAAGCCGGTAGATCAC | TCACATAGCAGTGGCATGTGCTG |
| *Sox21a-1* | ATGGCAAAGCCTATGGACC | TCATATGGTCGCAGCGTACGTC |
| *Sox21a-2* |  | TCATATCGCCGCAGCGTACGTC |
| *Sox21b-1* | ATGTCCAAGCCCATGGATCAC | TCATAACGCCGCCGCGTACGC |
| *Sox21b-2* | ATGTCCAAGTCTATGGATCAC |  |
| *Sox4a-1* | ATGGTGGAGCAGACGCACATCAGC | TCAGTAAGTAAACACCAAGTTGG |
| *Sox4a-2* |  | TCAATAAGTGAACACCAAGTTTG |
| *Sox4b-1* | ATGGTCCAGAAAACAAGCAGC | CTAGTTCAGCACGTGAGCCGTGAAC |
| *Sox4b-2* | ATGGTCCAGAAAACCAGCAGC |  |
| *Sox11a* | ATGGTGCAGCAAATGGACAACAGC | TCATTTCGCTGAGCTCAGGTGTG |
| *Sox11b* | ATGGTGCAGCAGACAGAGCACAGC | TCAGTAAGTGAAAACAAGGTC |
| *Sox12-1* | ATGACTCTAGGGAACCTGCTG | TTAGTAGGTGAACACAAGGTC |
| *Sox12-2* | ATGACTCTAGGGAACTTACTG | TTAGTAAGTGAACACTAGGTC |
| *Sox5-1* | ATGCTTACTGAGCCTGAGCTTCC | TTACTGAGTAATGTGATTCTC |
| *Sox5-2* |  | TTACTGAGTGATGTGATTCTC |
| *Sox6* | ATGTCTTCCAAGCAAGCCACCTC | TTAGATGGCACTGACCGGCTCC |
| *Sox13-1* | ATGTGTGAGCCCAGTCCACC | TCAATCAGTGAGGACAACCAGC |
| *Sox13-2* |  | TCAGTCAGTGAGGACGACCAGC |
| *Sox8a-1* | ATGTCAAAAGAGCCGGATAAAACTC | TCAGGGTCGTGTTAAAGTGGTG |
| *Sox8a-2* | ATGTCAAAAGAGCCTGATAAAACTC | TCAGGGTCGTGTTAACGTCGTG |
| *Sox8b-1* | ATGACGGACGAGCCCGAGAGGTC | TCAGGGTCTGGAGAGCGTGCTG |
| *Sox8b-2* | ATGACGGAGGAGCAGGAGAAGTC | TCACGGCCTGGATAACGTGGTG |
| *Sox9a* | ATGAATCTACTAGACCCCTACCT | TCAGGGTCTGGACAGCTG |
| *Sox9b-1* | ATGAATCTGTTCGACTCGTGCC | TCAGGGCCGGGACAGCTGGGTG |
| *Sox9b-2* | ATGAATCTCTTCGACTCCTTCC | TCAGTGCCTGGACAGCTGGGTG |
| *Sox10* | ATGTCGGCGGAGGAGCACAGTATG | TCATGGTCGAGATAAGGTGGTG |
| *Sox7* | ATGGCTGCTCTGATAAGCGCG | TTATGAAATGCTGTAGTTGTTG |
| *Sox17* | ATGAGCAGTCCCGATGCG | TCAGGAATTACTATAGCC |
| *Sox18-1* | ATGAATATATCTGAGTCTAGTTGC | TTATCCTGTAATGCACGCGC |
| *Sox18-2* |  | TTATCCCGTAATGCATGCGC |
| *Sox32* | ATGTATCTCGACCGGATGC | TCACTCGTTCTGGTCCACAGGC |

**Table S2.** Inventory of the *Sox* genes in BSB.

| Subfamily  group | Gene name | Length  (aa) | GenBank accession no. | Subfamily  group | Gene name | Length  (aa) | GenBank accession no. |
| --- | --- | --- | --- | --- | --- | --- | --- |
| B1 | BSB_*Sox1a* | 334 | MN723571 | D | BSB_*Sox5* | 505 | MN728114 |
|  | BSB_*Sox1b* | 339 | MN723578 |  | BSB_*Sox6* | 525 | MN728121 |
|  | BSB_*Sox2* | 316 | MN723585 |  | BSB_*Sox13* | 568 | MN728125 |
|  | BSB_*Sox3* | 300 | MN728043 | E | BSB_*Sox8a* | 407 | MN728132 |
|  | BSB_*Sox19a* | 297 | MN728050 |  | BSB_*Sox8b* | 448 | MN728139 |
|  | BSB_*Sox19b* | 293 | MN728057 |  | BSB_*Sox9a* | 459 | MN723570 |
| B2 | BSB_*Sox14* | 239 | MN728064 |  | BSB_*Sox9b* | 457 | MN728146 |
|  | BSB_*Sox21a* | 240 | MN728071 |  | BSB_*Sox10* | 488 | MN728153 |
|  | BSB_*Sox21b* | 248 | MN728078 | F | BSB_*Sox7* | 395 | MN728160 |
| C | BSB_*Sox4a* | 361 | MN728085 |  | BSB_*Sox17* | 348 | MN728167 |
|  | BSB_*Sox4b* | 379 | MN728092 |  | BSB_*Sox18* | 444 | MN728170 |
|  | BSB_*Sox11a* | 354 | MN728099 | K | BSB_*Sox32* | 289 | MN728177 |
|  | BSB_*Sox11b* | 364 | MN728103 |  |  |  |  |
|  | BSB_*Sox12* | 356 | MN728107 |  |  |  |  |

**Table S3.** Inventory of the *Sox* genes in 2nRCC.

| Subfamily  group | Gene name | Length  (aa) | GenBank accession no. | Subfamily  group | Gene name | Length  (aa) | GenBank accession no. |
| --- | --- | --- | --- | --- | --- | --- | --- |
| B1 | 2nRCC_*Sox1a*-1 | 337 | MN723572 | D | 2nRCC_*Sox5*-1 | 765 | MN728115 |
|  | 2nRCC_*Sox1a*-2 | 336 | MN723573 |  | 2nRCC_*Sox5*-2 | 755 | MN728116 |
|  | 2nRCC_*Sox1b*-1 | 343 | MN723579 |  | 2nRCC_*Sox6* | 769 | MN728122 |
|  | 2nRCC_*Sox1b*-2 | 336 | MN723580 |  | 2nRCC_*Sox13*-1 | 606 | MN728126 |
|  | 2nRCC_*Sox2*-1 | 316 | MN723586 |  | 2nRCC_*Sox13*-2 | 600 | MN728127 |
|  | 2nRCC_*Sox2*-2 | 316 | MN723587 | E | 2nRCC_*Sox8a*-1 | 390 | MN728133 |
|  | 2nRCC_*Sox3*-1 | 300 | MN728044 |  | 2nRCC_*Sox8a*-2 | 396 | MN728134 |
|  | 2nRCC_*Sox3*-2 | 298 | MN728045 |  | 2nRCC_*Sox8b*-1 | 390 | MN728140 |
|  | 2nRCC_*Sox19a*-1 | 297 | MN728051 |  | 2nRCC_*Sox8b*-2 | 427 | MN728141 |
|  | 2nRCC_*Sox19a*-2 | 297 | MN728052 |  | 2nRCC_*Sox9a*-1 | 443 | MK307773 |
|  | 2nRCC_*Sox19b*-1 | 293 | MN728058 |  | 2nRCC_*Sox9a*-2 | 466 | MK307791 |
|  | 2nRCC_*Sox19b*-2 | 290 | MN728059 |  | 2nRCC_*Sox9b*-1 | 420 | MN728147 |
| B2 | 2nRCC_*Sox14*-1 | 239 | MN728065 |  | 2nRCC_*Sox9b*-2 | 409 | MN728148 |
|  | 2nRCC_*Sox14*-2 | 239 | MN728066 |  | 2nRCC_*Sox10*-1 | 478 | MN728154 |
|  | 2nRCC_*Sox21a*-1 | 239 | MN728072 |  | 2nRCC_*Sox10*-2 | 483 | MN728155 |
|  | 2nRCC_*Sox21a*-2 | 231 | MN728073 | F | 2nRCC_*Sox7*-1 | 395 | MN728161 |
|  | 2nRCC_*Sox21b*-1 | 243 | MN728079 |  | 2nRCC_*Sox7*-2 | 398 | MN728162 |
|  | 2nRCC_*Sox21b*-2 | 243 | MN728080 |  | 2nRCC_*Sox17* | 433 | MN728168 |
| C | 2nRCC_*Sox4a*-1 | 353 | MN728086 |  | 2nRCC_*Sox18*-1 | 443 | MN728171 |
|  | 2nRCC_*Sox4a*-2 | 347 | MN728087 |  | 2nRCC_*Sox18*-2 | 440 | MN728172 |
|  | 2nRCC_*Sox4b*-1 | 371 | MN728093 | K | 2nRCC_*Sox32* | 307 | MN728178 |
|  | 2nRCC_*Sox4b*-2 | 377 | MN728094 |  |  |  |  |
|  | 2nRCC_*Sox11a* | 334 | MN728100 |  |  |  |  |
|  | 2nRCC_*Sox11b* | 359 | MN728104 |  |  |  |  |
|  | 2nRCC_*Sox12*-1 | 370 | MN728108 |  |  |  |  |
|  | 2nRCC_*Sox12*-2 | 370 | MN728109 |  |  |  |  |

**Table S4.** Inventory of the *Sox* genes in 4nRCC.

| Subfamily  group | Gene name | Length  (aa) | GenBank accession no. | Subfamily  group | Gene name | Length  (aa) | GenBank accession no. |
| --- | --- | --- | --- | --- | --- | --- | --- |
| B1 | 4nRCC_*Sox1a*-1 | 336 | MN723574 | C | 4nRCC_*Sox4b*-1 | 371 | MN728095 |
|  | 4nRCC_*Sox1a*-2 | 336 | MN723575 |  | 4nRCC_*Sox4b*-2 | 376 | MN728096 |
|  | 4nRCC_*Sox1a*-3 | 337 | MN723576 |  | 4nRCC_*Sox4b*-3 | 377 | MN728097 |
|  | 4nRCC_*Sox1a*-4 | 337 | MN723577 |  | 4nRCC_*Sox4b*-4 | 376 | MN728098 |
|  | 4nRCC_*Sox1b*-1 | 343 | MN723581 |  | 4nRCC_*Sox11a*-1 | 334 | MN728101 |
|  | 4nRCC_*Sox1b*-2 | 343 | MN723582 |  | 4nRCC_*Sox11a*-2 | 337 | MN728102 |
|  | 4nRCC_*Sox1b*-3 | 336 | MN723583 |  | 4nRCC_*Sox11b*-1 | 358 | MN728105 |
|  | 4nRCC_*Sox1b*-4 | 336 | MN723584 |  | 4nRCC_*Sox11b*-2 | 366 | MN728106 |
|  | 4nRCC_*Sox2*-1 | 316 | MN723588 |  | 4nRCC_*Sox12*-1 | 370 | MN728110 |
|  | 4nRCC_*Sox2*-2 | 316 | MN723589 |  | 4nRCC_*Sox12*-2 | 370 | MN728111 |
|  | 4nRCC_*Sox2*-3 | 316 | MN723590 |  | 4nRCC_*Sox12*-3 | 370 | MN728112 |
|  | 4nRCC_*Sox2*-4 | 316 | MN723591 |  | 4nRCC_*Sox12*-4 | 370 | MN728113 |
|  | 4nRCC_*Sox3*-1 | 300 | MN728046 | D | 4nRCC_*Sox5*-1 | 765 | MN728117 |
|  | 4nRCC_*Sox3*-2 | 300 | MN728047 |  | 4nRCC_*Sox5*-2 | 764 | MN728118 |
|  | 4nRCC_*Sox3*-3 | 298 | MN728048 |  | 4nRCC_*Sox5*-3 | 763 | MN728119 |
|  | 4nRCC_*Sox3*-4 | 301 | MN728049 |  | 4nRCC_*Sox5*-4 | 755 | MN728120 |
|  | 4nRCC_*Sox19a*-1 | 297 | MN728053 |  | 4nRCC_*Sox6*-1 | 769 | MN728123 |
|  | 4nRCC_*Sox19a*-2 | 297 | MN728054 |  | 4nRCC_*Sox6*-2 | 770 | MN728124 |
|  | 4nRCC_*Sox19a*-3 | 298 | MN728055 |  | 4nRCC_*Sox13*-1 | 600 | MN728128 |
|  | 4nRCC_*Sox19a*-4 | 298 | MN728056 |  | 4nRCC_*Sox13*-2 | 600 | MN728129 |
|  | 4nRCC_*Sox19b*-1 | 293 | MN728060 |  | 4nRCC_*Sox13*-3 | 603 | MN728130 |
|  | 4nRCC_*Sox19b*-2 | 293 | MN728061 |  | 4nRCC_*Sox13*-4 | 602 | MN728131 |
|  | 4nRCC_*Sox19b*-3 | 290 | MN728062 | E | 4nRCC_*Sox8a*-1 | 396 | MN728135 |
|  | 4nRCC_*Sox19b*-4 | 290 | MN728063 |  | 4nRCC_*Sox8a*-2 | 396 | MN728136 |
| B2 | 4nRCC_*Sox14*-1 | 239 | MN728067 |  | 4nRCC_*Sox8a*-3 | 390 | MN728137 |
|  | 4nRCC_*Sox14*-2 | 239 | MN728068 |  | 4nRCC_*Sox8a*-4 | 410 | MN728138 |
|  | 4nRCC_*Sox14*-3 | 239 | MN728069 |  | 4nRCC_*Sox8b*-1 | 390 | MN728142 |
|  | 4nRCC_*Sox14*-4 | 239 | MN728070 |  | 4nRCC_*Sox8b*-2 | 427 | MN728143 |
|  | 4nRCC_*Sox21a*-1 | 239 | MN728074 |  | 4nRCC_*Sox8b*-3 | 428 | MN728144 |
|  | 4nRCC_*Sox21a*-2 | 239 | MN728075 |  | 4nRCC_*Sox8b*-4 | 422 | MN728145 |
|  | 4nRCC_*Sox21a*-3 | 230 | MN728076 |  | 4nRCC_*Sox9a*-1 | 466 | MK307774 |
|  | 4nRCC_*Sox21a*-4 | 239 | MN728077 |  | 4nRCC_*Sox9a*-2 | 448 | MK307775 |
|  | 4nRCC_*Sox21b*-1 | 243 | MN728081 |  | 4nRCC_*Sox9a*-3 | 461 | MK307776 |
|  | 4nRCC_*Sox21b*-2 | 243 | MN728082 |  | 4nRCC_*Sox9a*-4 | 443 | MK307777 |
|  | 4nRCC_*Sox21b*-3 | 243 | MN728083 |  | 4nRCC_*Sox9b*-1 | 421 | MN728149 |
|  | 4nRCC_*Sox21b*-4 | 243 | MN728084 |  | 4nRCC_*Sox9b*-2 | 409 | MN728150 |
| C | 4nRCC_*Sox4a*-1 | 353 | MN728088 |  | 4nRCC_*Sox9b*-3 | 428 | MN728151 |
|  | 4nRCC_*Sox4a*-2 | 353 | MN728089 |  | 4nRCC_*Sox9b*-4 | 432 | MN728152 |
|  | 4nRCC_*Sox4a*-3 | 347 | MN728090 |  | 4nRCC_*Sox10*-1 | 478 | MN728156 |
|  | 4nRCC_*Sox4a*-4 | 343 | MN728091 |  | 4nRCC_*Sox10*-2 | 483 | MN728157 |
| Continued | | | | | | | |
| Subfamily  group | Gene name | Length  (aa) | GenBank accession no. | Subfamily  group | Gene name | Length  (aa) | GenBank accession no. |
| E | 4nRCC_*Sox10*-3 | 481 | MN728158 | F | 4nRCC_*Sox17* | 433 | MN728169 |
|  | 4nRCC_*Sox10*-4 | 483 | MN728159 |  | 4nRCC_*Sox18*-1 | 440 | MN728173 |
| F | 4nRCC_*Sox7*-1 | 398 | MN728163 |  | 4nRCC_*Sox18*-2 | 443 | MN728174 |
|  | 4nRCC_*Sox7*-2 | 398 | MN728164 |  | 4nRCC_*Sox18*-3 | 443 | MN728175 |
|  | 4nRCC_*Sox7*-3 | 395 | MN728165 |  | 4nRCC_*Sox18*-4 | 440 | MN728176 |
|  | 4nRCC_*Sox7*-4 | 395 | MN728166 | K | 4nRCC_*Sox32* | 307 | MN728179 |
